# Physiologically-Based Biopharmaceutics Modeling for Ibuprofen: Identifying Key Formulation Parameter and Virtual Bioequivalence Assessment

**DOI:** 10.3390/pharmaceutics17040408

**Published:** 2025-03-24

**Authors:** Javier Zarzoso-Foj, Marina Cuquerella-Gilabert, Matilde Merino-Sanjuan, Javier Reig-Lopez, Víctor Mangas-Sanjuán, Alfredo Garcia-Arieta

**Affiliations:** 1 Department of Pharmacy and Pharmaceutical Technology and Parasitology, University of Valencia, 46010 Valencia, Spain; 2 Interuniversity Research Institute for Molecular Recognition and Technological Development, Polytechnic University of Valencia, University of Valencia, 46100 Valencia, Spain; 3 Consultant for WHO Prequalification of Medicines Programme, 28080 Madrid, Spain

**Keywords:** ibuprofen, PBBM, PBPK, virtual bioequivalence

## Abstract

**Background**: Physiologically based pharmacokinetic (PBPK) modeling for biopharmaceutics applications (i.e., physiologically based biopharmaceutics modeling (PBBM)) enables mechanistic modeling from dissolution to absorption and disposition, facilitating the prediction of bioequivalence (BE) outcomes and the delimitation of the safe space. This study aims to identify the product-related parameter driving ibuprofen dissolution to upgrade an existing PBPK model, so that an in vitro safe space and virtual BE (VBE) predictions of IR ibuprofen tablets can be performed. **Methods**: C_max_ within- and between-subject variabilities of a previous PBPK model were optimized after identifying crucial physiological parameters for ibuprofen absorption and disposition. In vitro data modeling was performed to estimate the value of the parameter driving ibuprofen dissolution. A safe space was defined for this parameter and the sample size to declare BE was calculated. Finally, VBE simulations were performed to explore the effect of sample size as well as number of trial replicates and runs. **Results**: C_max_ variability was adequately predicted after changing V_ss_ and MRT in stomach and small intestine CV (%) to 10 and 150%, respectively. Particle surface pH was identified as the dissolution key parameter for ibuprofen. A safe space for test product surface pH values of 5.64–6.40 was defined in order to achieve a 90%CI for the C_max_ ratio within the 80–125% range when the reference product surface pH is 6.02. R-ibuprofen was identified as the most discriminative enantiomer. VBE studies with 24 individuals showed BE outcomes that are sensitive to the number of trial replicates and runs. **Conclusions**: Ibuprofen particle surface pH has been identified as the in vitro parameter governing dissolution in maleate buffer 7 mM with HCl pH 2.0 pretreatment, allowing to establish an in vitro safe space useful for calculating sample sizes and to evaluate the BE success rate through PBBM/PBPK model-informed VBE simulations.

## 1. Introduction

One of the most important challenges facing the development and authorization of medicines is to establish model-informed strategies that allow to rationally guide the design of new formulations or products and to anticipate their probability of success. Currently, the international regulatory framework encourages the use of model-informed drug discovery and development (MID3) strategies to justify the design of new pharmaceutical products, as well as post-approval changes [1,2,3,4,5]. The systematic use of these methodologies by the pharmaceutical industry and regulatory agencies facilitates more efficient and ethical development and authorization of generic medicines. This contributes to reducing the costs borne by national health systems, improving their long-term sustainability.

Physiologically based pharmacokinetic (PBPK) modeling has been consolidated as a powerful tool for predicting drug exposure in humans and other species [6,7,8,9]. By incorporating physiological parameters and mechanistic principles, PBPK models can provide valuable insights into the release, absorption, distribution, metabolism, and excretion (ADME) of drugs [10,11,12,13]. However, PBPK models often rely on limited in vivo data without information on the bioavailability of different formulations, which does not allow to capture the impact of formulation factors on drug absorption [4]. To address this limitation, physiologically based biopharmaceutics models (PBBMs) integrating in vitro dissolution data into PBPK frameworks have been developed to mechanistically characterize the drug release and dissolution through an in vitro–in vivo (IVIV) link [14,15,16,17]. Under this umbrella, the area of bioequivalence (BE) presents a promising future, since the factors involved in pharmacokinetic differences can be previously controlled and anticipated [18]. However, the main challenge is to develop and validate mathematical frameworks capable of integrating mechanistically the different factors involved in ADME processes. In this regard, several studies have been performed in order to establish clinically relevant drug product specifications [4], to identify safe spaces [19,20], and, ultimately, BE biowaivers through virtual BE (VBE) trials [21,22]. These practices emerge as promising methodologies to accelerate innovative and generic drug development due to their efficiency in the integration of knowledge generated throughout in vitro studies and clinical trials and support the regulatory approval to be leveraged for industry, national health systems, and patients.

Ibuprofen is a class IIa drug according to the Biopharmaceutics Classification System (BCS) and an optimal candidate for PBPK strategies as solubility and dissolution rate are critical for its absorption rate and, consequently, its onset of action [23,24]. Moreover, this molecule shows a remarkable self-buffering capacity, meaning that the pH around its particles is not the same as the pH of the gastrointestinal fluids, thus affecting its dissolution rate [25]. Finally, as it is a well-known and studied nonsteroidal anti-inflammatory drug with extensive preclinical and clinical data publicly available, ibuprofen is an excellent candidate for applying the above-mentioned in silico modeling strategies in order to demonstrate their potential in generics industry, anticipating BE outcomes in the fastest and safest way and, in the near future, requesting biowaivers regardless of the BCS class [26].

While existing PBPK models for ibuprofen can provide reliable predictions of drug exposure [27,28], further refinement to improve their predictive power is essential to consider the possibility of waiving in vivo BE studies of ibuprofen products. In this study, we aimed to develop a PBBM/PBPK framework for IR ibuprofen tablets able to (i) identify the key relevant in vitro parameter governing ibuprofen dissolution in biopredictive media, (ii) establish the in vitro safe space integrating external in vivo BE information with the existing PBPK model, and (iii) evaluate the BE success through VBE simulations.

## 2. Materials and Methods

**Figure 1:**
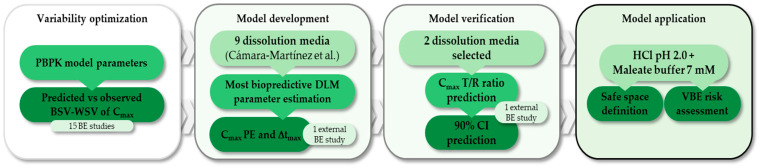
Workflow strategy followed [29]. PBPK: physiologically based pharmacokinetics; BSV: between-subject variability; WSV: within-subject variability; C_max_: maximum concentration; t_max_: time to maximum concentration; BE: bioequivalence; DLM: diffusion layer model; PE: prediction error: T/R: test/reference; CI: confidence interval; VBE: virtual bioequivalence.

An overall workflow is depicted in Figure 1. The Simcyp® In Vitro Data Analysis toolkit (SIVA, version 5, Certara UK Limited, Sheffield, UK) coupled with Simcyp® Simulator (version 21; Certara UK Limited) were used to model the in vitro dissolution data and to simulate the systemic exposure of ibuprofen enantiomers in healthy volunteers. The in vitro dissolution data and plasma concentration–time profiles reported in the literature [29] were extracted with Graphreader V2 (version 0.9, Jan 2023, Kristian P. Larsen www.graphreader.com, accessed on 10 June 2024).

### 2.1. PBPK Model Variability Optimization

An existing PBPK model for ibuprofen enantiomers [27] was used because of its ability to predict the systemic exposure of R- and S-ibuprofen after oral administration of IR dosage forms (solutions, suspensions, soft gelatin capsules, and tablets) of racemic ibuprofen. Briefly, the PBPK model considers the Advanced Dissolution, Absorption, and Metabolism (ADAM) model [13] together with stereoselectivity in plasma protein binding and CYP450-mediated metabolism, as well as the unidirectional R-to-S chiral inversion.

Default variability (CV (%)) on system- and drug-related parameters of the above-mentioned PBPK model was optimized using Simcyp® Simulator (v21R1) in order to predict the variability observed in C_max_ of 15 phase I clinical studies across different products, dosage forms, and dose levels of ibuprofen (Supplementary Materials Table S1). Between-subject variability (BSV) and within-subject variability (WSV) of C_max_ were evaluated following the methodology proposed by Bego et al. [18]. Concisely, this methodology assumes WSV to be the same as BSV in physiological parameters within a physiological plausible range. WSV was considered for mean residence times (MRTs) in the stomach and small intestine (SI), pH and volumes of the GI tract, and steady-state volume of distribution (V_ss_).

A global sensitivity analysis (GSA) using the Morris method [30] in Simcyp® Simulator was performed for selecting the PBPK parameters the model was most sensitive to and whose variability needed further optimization [31]. This methodology assesses the simultaneous change in parameters within a pre-established range and their impact on PK exposure endpoints. The assessment on how the PK outcomes change due to variations in model parameters was performed simultaneously without correlations between parameters. The parameter with the highest absolute mean (µ*) was selected for the optimization step.

### 2.2. PBBM Model Development and Verification

The SIVA toolkit allows to model biopharmaceutical in vitro data and estimate the parameters of the diffusion layer model (DLM) [32,33], which mechanistically describes the dissolution rate of drugs according to particle size, surface solubility (sensitive to particle surface pH), thickness of the hydrodynamic layer, and the concentration gradient in the diffusion layer. Experimental mean in vitro dissolution data of IR tablets containing 200 mg of racemic mixture of ibuprofen were collected from Cámara-Martínez et al. [29]. A summary of the characteristics of this experimental data set is provided in Supplementary Materials Table S2.

A GSA [30] was performed for selecting the PBBM parameters governing the solubility to be estimated (i.e., particle surface pH, solubility factor, intrinsic solubility scalar, and thickness of the diffusion layer) [34]. The assessment on how the PK outcomes change due to variations was performed simultaneously without correlations between parameters. A poly-dispersed particle size distribution was assumed. As in the previous GSA, the parameter with the highest absolute mean (µ*) was selected for the estimation step.

The estimation process was conducted for the selected parameter using the Nelder–Mead optimization algorithm. The non-selected parameters were assumed as equal between both products (reference and test). The selection criterion was based on the r^2^ value. Since only experimental information of ibuprofen as racemic mixture was available, DLM parameter values were assumed to be the same for both ibuprofen enantiomers.

Then, parameter estimates for each dissolution medium and product were used as inputs for the DLM coupled with the PBPK model (PBBM/PBPK framework). A single oral administration of 200 mg ibuprofen IR tablet to healthy volunteers in fasted state was simulated. Longitudinal PK profiles of racemic ibuprofen were generated for test and reference products and C_max_ levels were extracted for each in vitro dissolution media condition. The results from the PBBM/PBPK simulation step were compared with the observed C_max_ levels of racemic ibuprofen from a phase I clinical trial of 200 mg tablet product of ibuprofen that was not previously considered during the variability optimization step (EudraCT: 2017-002884-17 [29]). Simulations leading to prediction error (PE) on C_max_ for test and reference products within the highly demanding 0.9–1.11 range and ±30% on t_max_ were selected.

The PBBM/PBPK framework verification was performed by comparing the observed 90%CI of the C_max_ ratio and the BE outcome from the above-mentioned phase I clinical trial with the predictions obtained using the previously selected in vitro dissolution media [29]. Dissolution media leading to a more accurate prediction of the BE outcome were finally selected after simulating 1 run of 10 trial replicates of two-period, two-sequence crossover studies (n = 24, the sample size of the in vivo BE study).

### 2.3. PBBM/PBPK Framework Application

The verified PBBM/PBPK framework was used to generate an in vitro safe space for the most biopredictive dissolution media. DLM parameters of the reference product were fixed to the estimated values from SIVA. The DLM parameter selected during the GSA for the test product was changed in order to achieve 90%CI of C_max_ ratios ranging from 80.00 to 125.00%. Two-period, two-sequence crossover VBE studies (n = 24) of a single-dose regimen of 200 mg ibuprofen tablet in a fasted-state healthy volunteer population were performed for a predefined range of particle surface pH values for the test product. A sample size calculation was performed for each test product particle surface pH assessed in order to find the number of individuals needed for a successful BE study under 80 and 90% statistical power [35]. Based on the observed variability in the in vivo BE study (EudraCT: 2017-002884-17), upper and lower limits of the 90% confidence interval (CI) were calculated for each of the anticipated T/R ratios in the safe space.

Finally, VBE simulations (N = 100) with different numbers of subjects (n = 12, 24, or 36) and trial replicates (10, 25, 50) were performed. In order to allow a random generation of individuals, different seed numbers were considered in each run. Simulations consisted of a single oral administration of 200 mg IR ibuprofen tablets to virtual populations of healthy volunteers in fasted state. A virtual population with 50% female proportion and an age of subjects between 20 and 50 years was assumed. The sampling scheme used was 0, 0.33, 0.66, 1, 1.25, 1.5, 1.75, 2, 2.5, 3, 3.5, 4, 6, 8, 10, 12, 14, and 24 h after dosing, like in the clinical trial reported by Cámara-Martínez et al. [29]. The VBE success rate was calculated for each enantiomer (R- and S-) and racemic mixture of ibuprofen.

## 3. Results

### 3.1. PBPK Model Variability Optimization

The results from the GSA on absorption and disposition parameters revealed MRT and V_ss_ as the most impactful parameters on C_max_ for both enantiomers after oral administration of racemic ibuprofen, with a negligible impact of pH and volumes across all GI segments (Supplementary Materials Figure S1). Manual optimization of V_ss_ CV (%) to 10% was needed to properly describe ibuprofen enantiomers’ C_max_ variability after IV administration. CV (%) of fasted MRT for both fluids and fine particles in the stomach and small intestine (SI) were increased up to 150% for ibuprofen oral dosage forms. The variability of the other system- and drug-related parameters was kept as default, and those considered for WSV are shown in Table 1.

Table 2 summarizes observed versus predicted WSV and BSV in C_max_ after IV and oral administration of different dosage forms of racemic ibuprofen. Overall, observed and predicted WSV and BSV variabilities were in adequate agreement (mean relative error of BSV and WSV < 11%) across the different products and enantiomers. A slight trend of over-prediction of BSV and WSV is also observed.

### 3.2. PBBM Model Development and Verification

**Figure 2:**
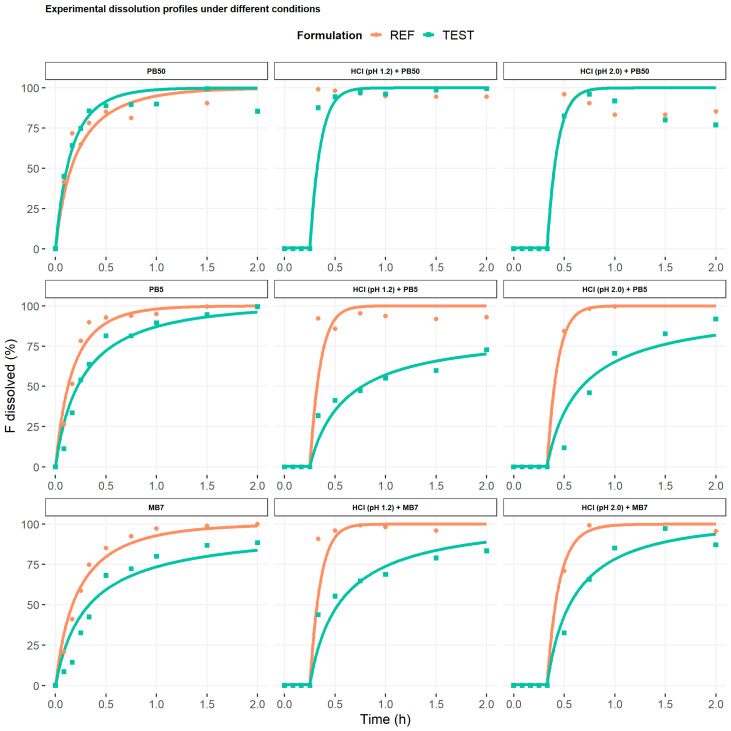
Individual fitting of mean dissolution data throughout different experimental conditions. Dots represent observed data and continuous lines predicted profile. PB50: phosphate buffer 50 mM; PB5: phosphate buffer 5 mM; MB7: maleate buffer 7 mM; F dissolved: fraction dissolved.

The results of the GSA on product-related parameters identified particle surface pH as the main parameter driving ibuprofen dissolution (Supplementary Materials Figure S2). Estimated particle surface pH values for reference and test products on the different dissolution media together with the PE on C_max_ and t_max_ after simulating a single 200 mg ibuprofen administration to a typical healthy volunteer are shown in Table 3. A satisfactory fitting of the experimental in vitro data was achieved in all cases (r^2^ > 0.90). Mean in vitro dissolution data together with predicted profiles can be found in Figure 2.

Manual fitting of particle surface pH was necessary for all experimental conditions with acidic pretreatment regardless of the product. PB5 without acidic pretreatment and MB7 with HCl pH 2.0 pretreatment were the in vitro dissolution media laying within the acceptance 0.90–1.11 range on C_max_ for both reference (1.01 and 1.09, respectively) and test (0.99 and 0.98, respectively) products. The t_max_ difference for the reference product considering MB7 with HCl pH 2.0 pretreatment was lower (−30%; 1.00 and 1.25 h being the observed and predicted values, respectively) compared to that of the PB5 without acidic pretreatment (−50%; 1.00 and 1.50 h being the observed and predicted values, respectively), while the t_max_ PE for the test product was equal for both dissolution media (−25%; 2.50 and 1.75 h being the observed and predicted values, respectively, for both media). According to the predefined acceptance criteria, MB7 with HCl pH 2.0 pretreatment was finally selected. Moreover, this dissolution medium was able to predict the failure in BE that occurred in the in vivo BE trial (90% CI C_max_: 80.48–93.21% and 79.69–91.18% for R- and S-ibuprofen, respectively), with 90% CI C_max_ of 77.22–83.65% and 78.90–85.25% for R- and S-ibuprofen, respectively.

### 3.3. PBBM Model Application

**Figure 3:**
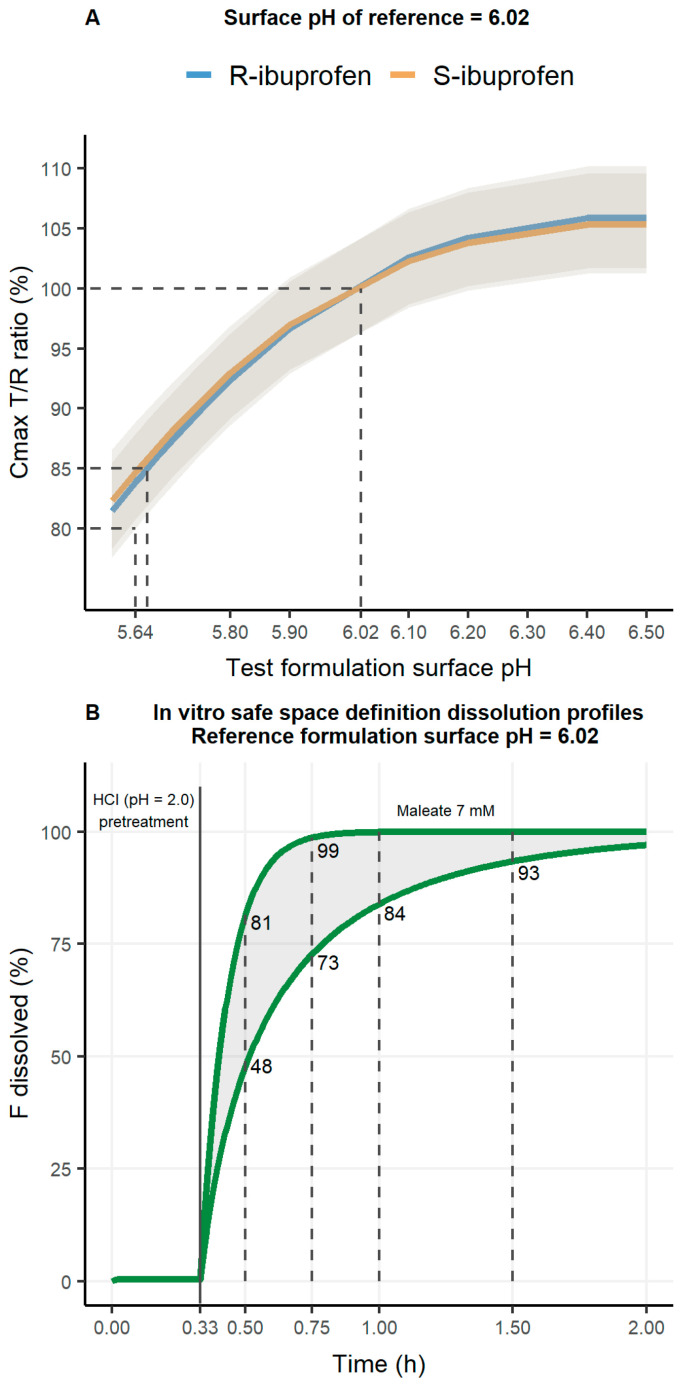
(**A**). Effect of particle surface pH on C_max_ T/R GMR (orange and blue solid lines) with the corresponding 90%CI (grey band) (**B**). Safe space for test products of immediate-release tablets of 200 mg racemic ibuprofen. T: test product; R: reference product.

Figure 3A shows the safe space identified for this medium (i.e., MB7 with HCl pH 2.0 pretreatment), where particle surface pH values of the test product of R-ibuprofen ranged from 5.64 (LL90%CI of C_max_ = 80.01%) to 6.40 (UL90%CI of C_max_ = 110.16%). The safe space considering particle surface pH for each enantiomer indicates an overlapping trend between enantiomers. Although the safe space was designed to cover the 80.00–125.00% range, the increase in particle surface pH greater than 6.40 does not increase the dissolution rate. Despite this, R-ibuprofen is slightly more discriminative against particle surface pH. Moreover, the greater the difference in the particle surface pH between test and reference products, the greater the differences between the C_max_ GMR of enantiomers. Consequently, in vitro dissolution profiles within the predicted safe space (particle surface pH between 5.64 and 6.40) for C_max_ were generated (Figure 3B). After HCl pretreatment at pH = 2.0, the dissolution rate should be rapid (fraction dissolved 48 to 81% and 73 to 99% at 0.5 and 0.75 h, respectively). Complete (100%) dissolution is predicted to be achieved at 0.75 h and >2 h with the particle surface pH 6.40 and 5.64, respectively.

Following the trend observed in the safe space depicted in Figure 3A, the higher the difference between particle surface pH between products, the larger the expected difference between products and the higher the sample size of the clinical trial needed to guarantee BE success with enough statistical power (80 or 90%) (Table 4) [35]. This higher sample size is amplified also because the observed WSV for the R-enantiomer was slightly higher than that for the S-enantiomer (14.89% vs. 13.65%). This higher variability for the R-enantiomer was also observed in Table 2 for tablets (14.90% vs. 12.15%), but the predicted ones in Table 2 were in the contrary order (15.83% vs. 16.60%). For products leading to an 85% C_max_ T/R GMR, and assuming the WSV observed in the in vivo study, sample sizes of 74 and 104 (R-ibuprofen) and 64 and 88 (S-ibuprofen) subjects are necessary to achieve 80 and 90% statistical power, respectively. On the other hand, scenarios with C_max_ T/R GMR of 95% would need only 10 and 14 (R-ibuprofen) and eight and 12 (S-ibuprofen) subjects to achieve 80 and 90% statistical power, respectively.

**Figure 4:**
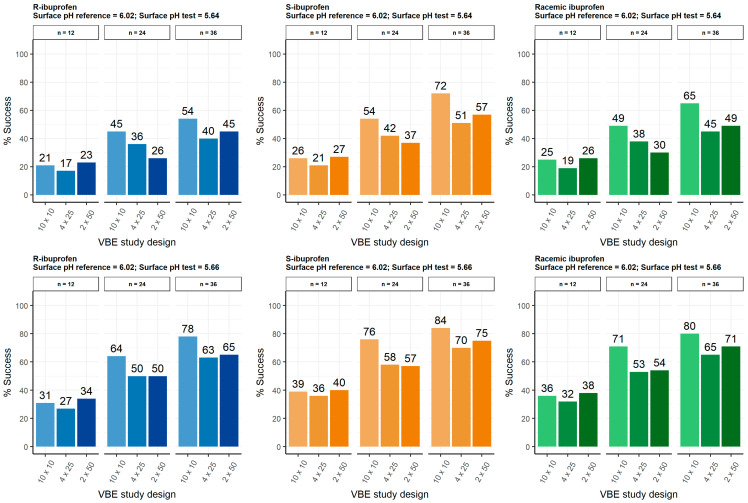
VBE success rate for ibuprofen enantiomers and racemic mixture under different sample size, test formulation particle surface pH of R-ibuprofen, and number of runs and trial replicates.

The PBBM/PBPK framework was finally used to assess in silico the effect of test product particle surface pH, sample size, and trial replicates on BE outcomes of immediate-release 200 mg ibuprofen tablets for both enantiomers and the racemic mixture. Figure 4 illustrates the percentage of successful trials in each scenario after VBE testing considering the BSV and WSV previously optimized with 15 phase I clinical trials. R-ibuprofen being more discriminative and slightly less variable in the predictions than its S-enantiomer (15.83% vs. 16.60%), the VBE trials showed lower chances of BE for R-ibuprofen regardless of the scenario considered (test formulation particle surface pH, study design, and sample size). This is also endorsed by the higher sample size needed for the same surface pH between enantiomers in the standard sample size calculation (see Table 4, surface pH 5.64 and 5.66), but in this case the variability employed in the calculations is the WSV of the in vivo study, which is higher for the R-enantiomer, which explains why the sample size is larger when the point estimates are similar for both enantiomers with the test product particle surface pH between 5.70–5.84.

Regarding surface pH, the assessment was firstly performed assuming a test product with a particle surface pH of 5.64 that leads to an LL 90% CI C_max_ ratio of 80% for R-ibuprofen. Success rates increased with sample size because of narrowing of the 90% CI, but no clear trend was observed between study designs. For example, when n = 12, the success rate is approximately 20% for all scenarios, whilst for n = 24 a decreasing trend when increasing the number of trial replicates per run (10 × 10 vs. 4 × 25 vs. 2 × 50) was observed. Similar behavior was observed when increasing particle surface pH for the test product to 5.66 (C_max_ GMR of R-ibuprofen = 85%), with a higher success rate in all scenarios (see Figure 4). These results were surprising, since the number of simulations is equivalent between the designs (N = 100) and different seeds have been considered in each run. On the other hand, when performing VBE simulations with a test formulation surface pH of 5.66, a success rate of 80% was achieved in 10 × 10 simulations concerning 36 subjects, while a conventional sample size calculation suggested more than 60 subjects to achieve enough power of BE. These results suggested that the VBE success rate is overestimated compared to the conventional sample size calculation.

## 4. Discussion

Model-based analysis of in vitro biopharmaceutic data has an important role in complementing the predictive capacity of a PBPK model, as it goes beyond the classical input of experimental dissolution data (percent dissolved vs. time) and allows to simulate in vivo drug dissolution in each individual of a virtual population. In this work, physiologically based biopharmaceutic modeling (PBBM) has been used to couple in vitro dissolution data of ibuprofen with an existing physiologically based pharmacokinetic (PBPK) model of ibuprofen enantiomers, generating a sufficiently predictive mechanistic framework that allows to perform credible VBE assessments in order to anticipate the bioavailability of different immediate-release tablets of racemic ibuprofen. Its regulatory use for biowaivers would require an even better prediction of the point estimates and WSV, including different dosage forms and strengths, to confirm its prediction capacity.

This study focused on C_max_ and t_max_. AUC was not assessed because BE demonstration for ibuprofen AUC is not problematic since it is completely absorbed independently of the absorption rate and its WSV variability is smaller than that of C_max_ [36].

The PBBM/PBPK framework used in this work was developed in an increasing-complexity modeling process. Firstly, variability of PBPK parameters was optimized based on 15 phase I clinical trials, being able to predict BSV and WSV variability components for C_max_ accurately, with mean predicted values of 17 and 21% (15 and 21% observed) for WSV and BSV, respectively. All products were predicted with similar accuracy, except soft gelatin capsules. This could be explained by the fact that the specific composition of the capsules used in the clinical study was not completely known, thus impairing the parameterization of the DLM of this product as differences in the physical state of ibuprofen inside the soft gelatin capsules may impact PK exposure parameters or because ibuprofen is dissolved inside the capsule and it precipitates in the stomach due to its acidic pH. It is noteworthy that the slight over-prediction of C_max_ variability observed in Table 2 (15.83% and 16.60% for R- and S-ibuprofen) generates a conservative framework that allows to perform more cautious BE risk assessments. However, the simulations of this in vivo BE study have shown a much lower WSV, based on the width of the 90%CI, which cannot be explained. The in vivo BE trial reported CV = 14.89% and 13.65% for R- and S-ibuprofen, respectively, and the simulated study predicted 8.08% and 7.82% for R- and S-ibuprofen, respectively. In this case the simulations are only more conservative because the simulated GMR identifies a larger difference.

Secondly, in vitro dissolution media of IR tablets containing 200 mg of ibuprofen were characterized through a PBBM model developed in SIVA. The DLM model mechanistically describes the dissolution process of immediate-release oral products, allowing to deconvolute the dissolution process into fundamental parameters such as particle size, intrinsic solubility, and particle surface pH, which can separately be evaluated by modeling in vitro dissolution data. The GSA identified particle surface pH as the driving factor governing the solubility of ibuprofen, since this parameter merges the physicochemical properties of the drug, the excipients present in the product, as well as in vitro dissolution conditions [28,37,38]. The relevance of particle surface pH on in vivo ibuprofen dissolution is in line with previous reports integrating this parameter into mechanistic oral absorption models [39]. Moreover, Bermejo et al. also needed to account for particle surface pH in addition to product particle size distribution to properly describe the ibuprofen absorption rate and establish an IVIV prediction [26]. In our work, by keeping all other parameters of the DLM as default, the particle surface pH was estimated for the reference and test products of the racemic mixture of ibuprofen at different experimental conditions. The adequate prediction of the in vitro dissolution profiles solely by estimating the particle surface pH for each product demonstrates the validity of the DLM model to accommodate different experimental conditions (i.e., with and without acidic pretreatment).

Thirdly, the integration of the dissolution information on the PBPK model was finally conducted. The estimated particle surface pH values together with the rest of the DLM parameters (fixed between products) were coupled with the existing PBPK model for ibuprofen IR tablets. This PBPK model allows to simulate and evaluate each ibuprofen enantiomer separately, which could be combined into the racemic mixture afterwards. Moreover, the model considered the unidirectional R-to-S chiral inversion by means of an intrinsic clearance mediated by a cytosolic racemase, simplifying the complex sequential enzymatic reactions that are involved in this process. Therefore, C_max_ and t_max_ for R- and S-ibuprofen after an IR 200 mg ibuprofen tablet were predicted assuming the same study design conditions as reported in the phase I clinical trial [29]. This analysis confirmed the biopredictive performance (PE within the 0.9–1.11 range for C_max_) of PB5 without acidic pretreatment and MB7 with HCl pH 2.0 pretreatment media for test and reference products, as the in vivo data from this study were not previously considered during the variability optimization step. Differences between observed and predicted t_max_ values indicated higher accuracy in predicting the ibuprofen absorption rate for MB7 with HCl pH 2.0 pretreatment, with differences of 30% and 25% for reference and test products, respectively.

Fourthly, prediction of the 90%CI for the C_max_ and BE outcome of the previously selected media (i.e., MB7 with HCl pH 2.0 pretreatment) further supported its selection over PB5 without acidic pretreatment for safe space design and VBE simulations, as it anticipated the BE failure reported by the in vivo study. Despite the C_max_ GMR predicted with MB7 with HCl pH 2.0 pretreatment being lower than observed (80.37 vs. 86.61 for R-ibuprofen and 82.02 vs. 85.24 for S-ibuprofen), the ability of this medium to anticipate BE failure positions itself as a conservative approach to perform VBE assessments, although the WSV is under-predicted in the simulations of the in vivo study.

Cámara-Martinez et al. identified MB7 with HCl pH 1.2 pretreatment and PB5 with HCl pH 2.0 pretreatment as the most biopredictive media for a Level A IVIVC [29]. Differences in C_max_ were higher than those predicted in this work, as the IVIVC strategy is based on the use of the Weibull model (empirical) for the characterization of the dissolution profiles and the use of the in vivo profiles to adjust the empirical in vitro–in vivo function. In our work, the in vivo data have not enriched the PBPK model; indeed, they have acted as an external validation of the PBBM/PBPK framework. This represents an advance in the capacity for reusing and the interchangeability of models for drug development.

When establishing a safe space to set drug product specifications based on the associated systemic exposure, the extreme batches have to be bioequivalent (i.e., acceptance range of 90.00–111.11% between the mean batch and the extreme batches or 80.00–125% between extreme batches). However, when the safe space is used to define the generic product performance that ensures BE, the acceptance range for the 90%CI of the GMR T/R is 80.00–125% and that should also be an extreme of the specifications of the generic product that ensures bioequivalence of all future batches of the generic product with respect to the bio-batch of the reference product. The safe space was defined solely for MB7 with the HCl pH 2.0 pretreatment condition, showing that, for reference products with particle surface pH of 6.02, test product particle surface pH could have values between 5.64–6.40 for R-ibuprofen to obtain a C_max_ ratio 90%CI of 80–125% (see Figure 3). The safe space definition states that R-ibuprofen is slightly more sensitive to particle surface pH changes, thus positioning it as the most discriminative analyte. These findings are in line with previous reports [27], where R-ibuprofen was identified as the most sensitive analyte to perform a BE failure risk assessment of oral suspensions containing racemic ibuprofen. This impacts sample size calculation since for a hypothetical test product with a particle surface pH value of 5.70, R-ibuprofen would need 17% more participants enrolled than S-ibuprofen to guarantee BE success. However, it has to be taken into account that the same physicochemical properties as well as dissolution rate have been considered for both enantiomers, indicating that the kinetic differences between the analytes are the cause of the different VBE outcomes for IR 200 mg ibuprofen tablets, as equal dissolution rates between enantiomers were considered.

Finally, the VBE simulations with different sample sizes and study design conditions (10 × 10, 4 × 25, and 2 × 50) showed an overestimation of the BE success. This seems to be caused by an under-prediction of the WSV, although it could be a consequence of a limited number of simulations (N = 100) or a biased generation of random individuals based on the BSW and WSV in the model parameters. Although the most common design is only one run of 10 trial replicates (1 × 10) [40,41,42], the results of this analysis showed no coherence between the number of runs and trial replicates in the VBE success rate (see Figure 4). On the other hand, racemic ibuprofen showed intermediate success rates between ibuprofen enantiomers in all scenarios, as expected. Therefore, the use of achiral bioanalytical methods for ibuprofen quantification can lead to incorrect control of type II error in BE tests, since the racemic mixture could not demonstrate BE because of the pharmacologically inactive moiety (R-ibuprofen), while it would be for the eutomer (S-ibuprofen). Additionally, the use of achiral bioanalytical methods (racemic mixture) leads to a conservative control of type I error for true non-BE formulations.

Despite the accuracy in predicting ibuprofen enantiomers exposure and the approximation of BE outcomes of our PBBM/PBPK framework, the model exhibits some limitations. In the first place, analytical errors could not be incorporated into VBE simulations; therefore, they were not considered in the variability optimization step. In vitro dissolution data modeling was performed with mean dissolution profiles and the same particle size distribution was considered for both reference and test products. Finally, ibuprofen particles surface pH was assumed to be constant throughout the GI tract.

## 5. Conclusions

In conclusion, a PBBM/PBPK framework has been successfully developed for IR 200 mg ibuprofen tablets. Particle surface pH has been identified as the in vitro parameter governing dissolution of ibuprofen in MB7 with pH 2.0 pretreatment, allowing to predict the average relative bioavailability of a generic product with a slight over-prediction of the point estimate differences and under-prediction of the WSV and to establish an in vitro safe space, which is useful for calculating the sample size of bioequivalence studies of other generic products with conventional sample size calculations because the BE success rate obtained through PBBM/PBPK model-informed VBE simulations seems to be inflated. Thus, additional research should be conducted to fully understand the differences observed.

## Figures and Tables

**Table 1:** Parameters selected for within-subject variability.

Selected Parameter	Variation (CV (%))	Minimum Limit	Parameter Value	Maximum Limit
Fasted MRT stomach fluid *	150	0.01	0.12	12
Fasted MRT SI fluid *	150	0.5	3.4	12
pH fasted duodenum	16	0	6.4	15
pH fasted jejunum 1	13	0	6.5	15
pH fasted jejunum 2	11	0	6.6	15
pH fasted ileum 1	10	0	6.8	15
pH fasted ileum 2	10	0	7	15
pH fasted ileum 3	7	0	7.1	15
pH fasted ileum 4	6	0	7.3	15
pH fasted colon	13	3.18	6.6	9.8
Initial volume of stomach fluid fasted	30	20	50	1000
Total Jej1 Ile4 volume fasted	30	10	105	1000
Colon volume fasted	0	1	13	250
V_ss_ (user input) treatment 1 *	10	0.05	0.091249	1000
V_ss_ (user input) treatment 2 *	10	0.05	0.091249	1000
Fasted MRT stomach fine particles treatment 1 *	150	0.01	0.27	12
Fasted MRT SI fine particles treatment 1 *	150	0.5	3.4	12
Fasted MRT stomach fine particles treatment 2 *	150	0.01	0.27	12
Fasted MRT SI fine particles treatment 2 *	150	0.5	3.4	12

MRT: mean residence time. SI: small intestine; Jej: jejunum; Ile: ileum. * Parameters with optimized variability.

**Table 2:** Observed and predicted variabilities of C_max_ for each ibuprofen enantiomer and dosage form.

Variability	Enantiomer	IV		Solution		Suspension		Soft Gelatin Capsules		Tablets	
		Obs	Pred	Obs	Pred	Obs	Pred	Obs	Pred	Obs	Pred
WSV	R	15.63	16.73	15.63	19.73	14.25	14.65	16.05	15.55	14.90	15.83
	S	16.93	17.98	16.93	20.05	13.35	14.95	16.00	15.55	12.15	16.60
BSV	R	17.05	16.63	22.03	23.68	25.10	23.30	18.20	23.80	21.90	19.93
	S	16.23	19.43	19.38	24.55	22.30	22.70	18.35	23.35	20.40	21.67

IV: intravenous administration; Solution: oral solution with arginine; Obs: observed variability; Pred: predicted variability; WSV: within-subject variability; BSV: between-subject variability; R: R-ibuprofen; S: S-ibuprofen.

**Table 3:** Estimated particle surface pH for reference and test products at different experimental in vitro conditions and the corresponding C_max_ prediction error and t_max_ difference (%).

Pretreatment	Treatment		Reference Product				Test Product			
	Medium	pH	Particle Surface pH	r^2^	C_max_ PE	Δ t_max_ (%)	Particle Surface pH	r^2^	C_max_ PE	Δ t_max_ (%)
None	PB50	6.8	5.71	0.93	0.96	50	5.85	0.95	1.14	−40
HCl (pH 1.2)	PB50	6.8	6.29 *	0.91	1.13	25	6.25 *	0.95	1.24	−50
HCl (pH 2.0)	PB50	6.8	6.30 *	0.94	1.13	25	6.20 *	0.95	1.24	−50
**None**	**PB5**	**6.7**	**5.81**	**0.97**	**1.01**	**50**	**5.58**	**0.96**	**0.99**	**−30**
HCl (pH 1.2)	PB5	6.7	6.28 *	0.92	1.13	25	5.30 *	0.96	0.80	−7
HCl (pH 2.0)	PB5	6.7	6.25 *	1.00	1.12	25	5.42 *	0.95	0.89	−20
None	MB7	6.5	5.64	0.97	0.93	75	5.40	0.91	0.87	−20
HCl (pH 1.2)	MB7	6.5	6.33 *	0.94	1.13	25	5.49 *	0.95	0.93	−20
**HCl (pH 2.0)**	**MB7**	**6.5**	6.02 *	**0.99**	**1.09**	**25**	5.57 *	**0.98**	**0.98**	**−30**

r^2^: linear correlation coefficient; PE: prediction error; PB50: phosphate buffer 50 mM; PB5: phosphate buffer 5 mM; MB7: maleate buffer 7 mM; * Manual fitting.

**Table 4:** Sample size calculation for each enantiomer and test product particle surface pH.

Enantiomer	Test Product Particle Surface pH	Predicted	Calculated Sample Size		Calculated 90% CI	
		C_max_ GMR	80% Power	90% Power	LL	UL
R-ibuprofen	5.64	84.00	114	158	81.31	86.78
R-ibuprofen	5.66	85.00	74	104	81.62	88.52
R-ibuprofen	5.70	87.00	40	54	82.28	92.00
R-ibuprofen	5.75	90.00	20	28	82.98	97.61
R-ibuprofen	5.80	93.00	12	18	83.35	103.77
R-ibuprofen	5.85	95.00	10	14	83.99	107.45
S-ibuprofen	5.64	85.00	64	88	81.66	88.48
S-ibuprofen	5.66	86.00	44	62	81.91	90.29
S-ibuprofen	5.70	87.00	34	46	82.28	91.99
S-ibuprofen	5.73	90.00	18	24	83.16	97.40
S-ibuprofen	5.80	93.00	12	14	84.11	102.84
S-ibuprofen	5.84	95.00	8	12	83.25	108.41

90%CI: 90% confidence interval assuming the WSV observed in the BE study and the sample size required to reach at least 80% power. LL: lower limit; UL: upper limit; GMR: geometric mean ratio. Particle surface pH for reference product was 6.02.

## Data Availability

No new data were created.

## Supplementary Materials

The following supporting information can be downloaded at https://www.mdpi.com/article/10.3390/pharmaceutics17040408/s1: Table S1: Summary of phase I clinical trial reports; Table S2: Summary of dissolution media tested. Extracted from Cámara-Martínez et al.; Figure S1: Results from GSA on PBPK parameters for each enantiomer; Figure S2: Results from the GSA on DLM parameters for each enantiomer.

## Abbreviations

The following abbreviations are used in this manuscript:

ADAM	Advanced Dissolution, Absorption, and Metabolism
ADME	Absorption, distribution, metabolism, and excretion
BCS	Biopharmaceutics Classification System
BE	Bioequivalence
BSV	Between-subject variability
CI	Confidence interval
C_max_	Maximum concentration
CV (%)	Coefficient of variation
DLM	Diffusion layer model
GI	Gastrointestinal
GSA	Global sensitivity analysis
IV	Intravenous
IVIV	In vitro–in vivo
MB7	Maleate buffer 7 mM
MRT	Mean residence time
PB5	Phosphate buffer 5 mM
PB50	Phosphate buffer 50 mM
PBBM	Physiologically based biopharmaceutics model(ing)
PBPK	Physiologically based pharmacokinetic
PE	Prediction error
PK	Pharmacokinetic
SI	Small intestine
SIVA	SimCyp® In Vitro Data Analysis toolkit
T/R	Test/reference
VBE	Virtual bioequivalence
V_ss_	Steady-state volume of distribution
WSV	Within-subject variability
