# Supplementary material for: Physiologically-Based Biopharmaceutics Modeling for Ibuprofen: Identifying Key Formulation Parameter and Virtual Bioequivalence Assessment
Source: Pharmaceutics. 2025 Mar 24;17(4):408. doi: 10.3390/pharmaceutics17040408 (PMC12030207; doi:10.3390/pharmaceutics17040408)
Supplement: Supplementary file 1 [file pharmaceutics-17-00408-s001.zip › pharmaceutics-3526833-supplementary.pdf]

Physiologically-Based Biopharmaceutics Modeling for Ibuprofen: identifying key formulation parameter and virtual bioequivalence assessment

Javier Zarzoso-Foj, Marina Cuquerella-Gilabert, Matilde Merino-Sanjuán, Javier Reig-López, Alfredo García-Arieta and Víctor Mangas-Sanjuán

Supplementary material

Table S1. Summary of Phase I clinical trial reports.

Study	Reference product		Test product		N (female%)	Age	Dose (mg)	Observations
	RoA	Formulation	RoA	Formulation				
**1**	ORAL	Solution	ORAL	Solution	24 (50)	19-31	600	Arginine salt for ibuprofen oral solutions
**2**	ORAL	Solution	ORAL	Solution	24 (50)	19-52	600	Arginine salt for ibuprofen oral solutions
**3**	ORAL	Solution	IV	Parenteral solution	24 (33.3)	18-29	400	Arginine salt for ibuprofen oral solutions
**4**	ORAL	Solution	IV	Parenteral solution	35 (48.6)	18-32	400	Arginine salt for ibuprofen oral solutions
**5**	ORAL	Suspension	ORAL	Suspension	14 (0)	19-38	200	
**6**	ORAL	Suspension	ORAL	Suspension	24 (54)	19-40	400	
**7**	ORAL	Soft gelatine capsules	ORAL	Soft gelatine capsules	25	19-59	400	Lysine salt for ibuprofen capsules
**8**	ORAL	Soft gelatine capsules	ORAL	Soft gelatine capsules	27 (51.9)	20-56	400	
**9**	ORAL	Tablet	ORAL	Tablet	29 (0)	18-43	600	
**10**	ORAL	Tablet	ORAL	Tablet	24 (50)	19-30	600	
**11**	ORAL	Tablet	ORAL	Tablet	24 (66.7)	18-60	600	
**12**	ORAL	Tablet	ORAL	Suspension	24 (50)	18-52	400	
**13**	ORAL	Effervescent tablet	ORAL	Tablet	34 (52.9)	19-51	400	
**14**	ORAL	Solution	ORAL	Solution	23 (47.8)	21-30	200	
**15**	ORAL	Tablet	ORAL	Capsule	36 (0)	19-43	200	

RoA: route of administration; N: sample size; IV: intravenous.

Table S2. Summary of dissolution media tested. Extracted from Cámara-Martínez et al.

DISSOLUTION MEDIA	PRETREATMENT			TREATMENT			
	COMPOSITION	pH	CONDITIONS	COMPOSITION		pH	CONDITIONS
1	-	-	-	Phosphate	50 mM	6.8	500 mL, 50 rpm
2	-	-	-	Phosphate	5 mM	6.7	500 mL, 50 rpm
3	-	-	-	Maleate	7 mM	6.5	500 mL, 50 rpm
4	HCl	1.2	15', 20 mL, 50 rpm	Phosphate	50 mM	6.8	500 mL, 50 rpm
5	HCl	1.2	15', 20 mL, 50 rpm	Phosphate	5 mM	6.7	500 mL, 50 rpm
6	HCl	1.2	15', 20 mL, 50 rpm	Maleate	7 mM	6.5	500 mL, 50 rpm
7	HCl	2	20', 20 mL, 50 rpm	Phosphate	50 mM	6.8	500 mL, 50 rpm
8	HCl	2	20', 20 mL, 50 rpm	Phosphate	5 mM	6.7	500 mL, 50 rpm
9	HCl	2	20', 20 mL, 50 rpm	Maleate	7 mM	6.5	500 mL, 50 rpm

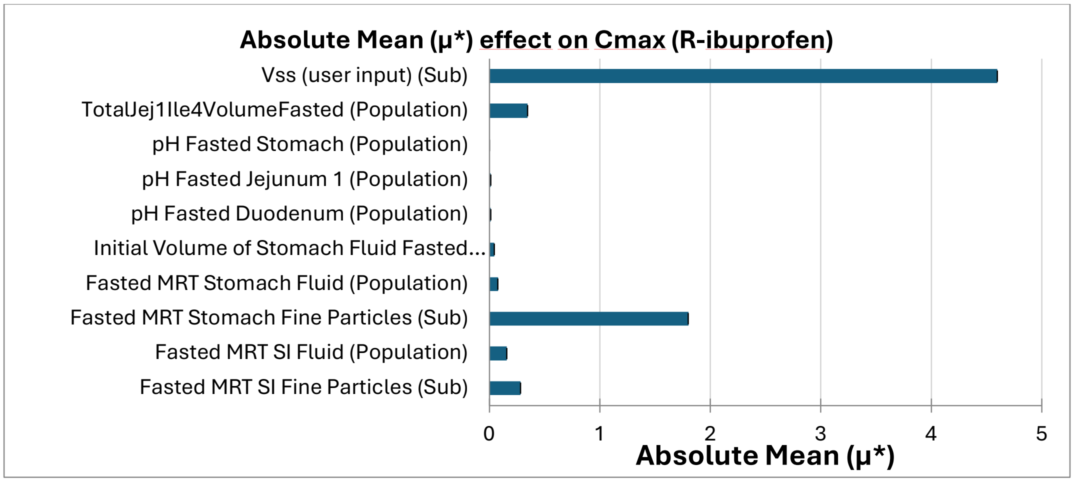

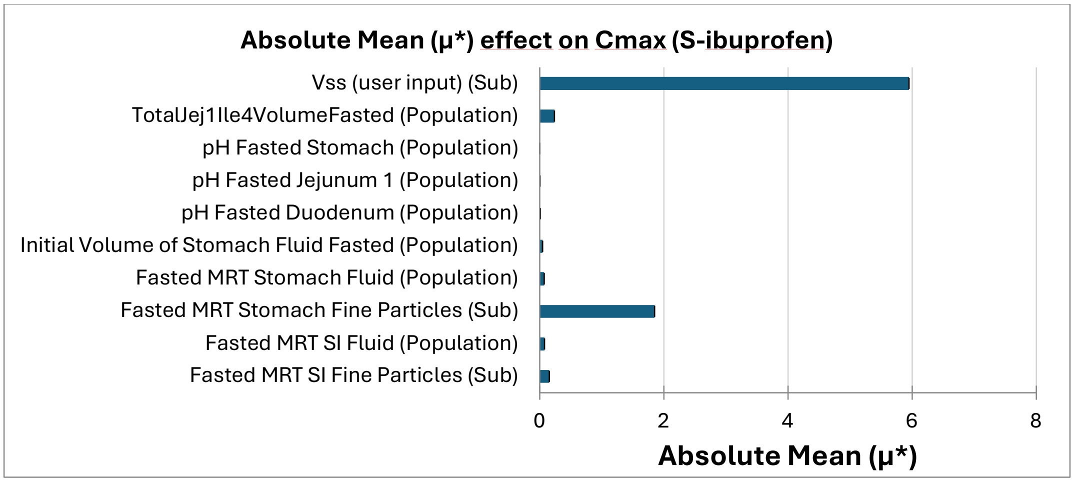

Figure S1. Results from the GSA analysis on PBPK parameters for each enantiomer. Vss: steady-state volume of distribution; MRT: mean residence time; SI: small intestine.

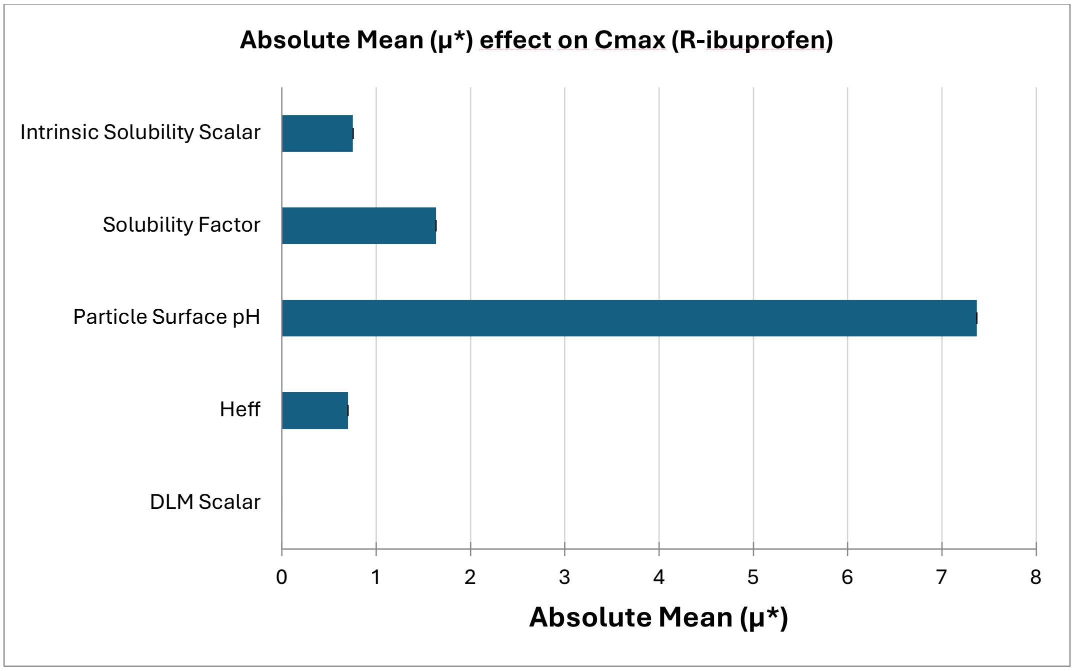

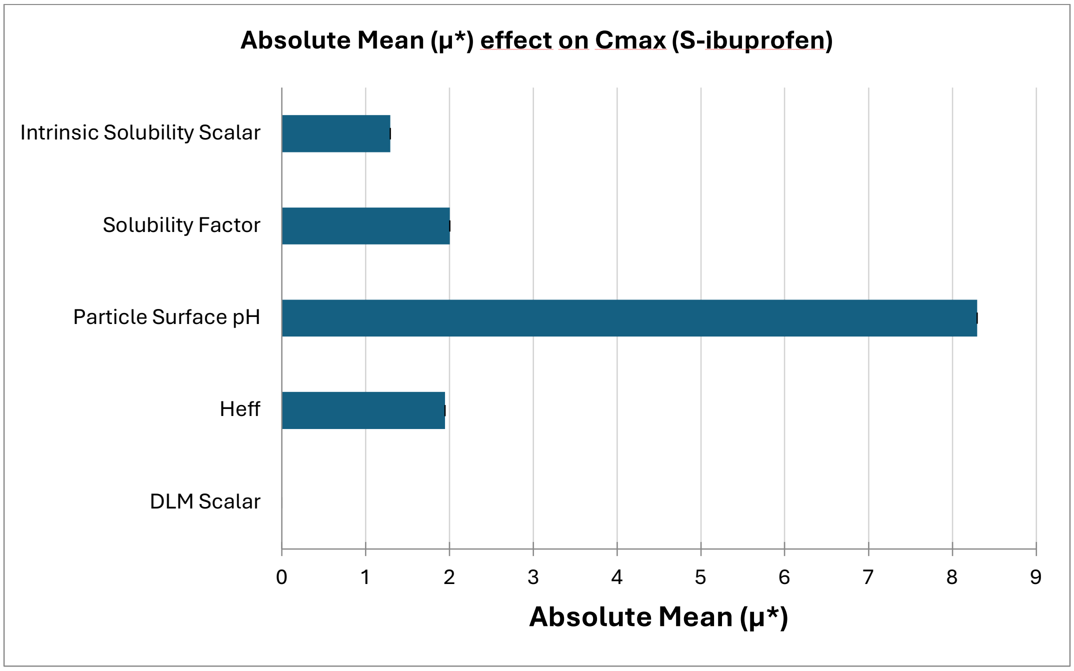

Figure S2. Results from the GSA analysis on DLM parameters for each enantiomer. Heff: Effective diffusion layer thickness; DLM: diffusion layer model.
